# Foot Health and Lower Extremity Function in People With Multiple Sclerosis: A Cross‐Sectional Survey Study

**DOI:** 10.1002/jfa2.70086

**Published:** 2025-09-18

**Authors:** Minna Stolt, Maria Eränen, Jouko Katajisto, Riitta Rosio

**Affiliations:** ^1^ Department of Nursing Science University of Turku Turku Finland; ^2^ The Wellbeing Services County of Satakunta Pori Finland; ^3^ Department of Statistics and Mathematics University of Turku Turku Finland; ^4^ Satakunta University of Applied Sciences Pori Finland

**Keywords:** foot health, lower extremity, multiple sclerosis, rehabilitation, survey

## Abstract

**Introduction:**

Foot health and lower extremity function are important issues for people with multiple sclerosis (MS). However, relatively little is known about foot health among people with MS. The potential association between foot health and lower extremity function in particular has seldom been studied. Therefore, this study aimed to analyse the level of self‐reported foot health and lower extremity function in people with MS and to identify possible associating factors.

**Methods:**

A cross‐sectional survey study design was applied. The data were collected online April–May 2024 from members of a national patient association with the Self‐administered Foot Health Assessment Instrument, the Lower Extremity Functional Scale (LEFS) as well as a background information form. The data were analysed with descriptive and inferential statistics.

**Results:**

The participants (*n* = 969, response rate 23%) had many foot problems of which dry skin (73%), cold feet (65%), leg cramps (61%), foot pain (59%) and thickened toe nails (51%) were the most common. Participants experienced mild to moderate lower extremity‐related functional limitation (mean 51, SD 22, range 0–80). Foot health among people with MS associated with gender, being on sick leave due to foot problems, perceived knowledge levels of foot self‐care, and self‐evaluated level of foot health. Moreover, weak but significant correlation between foot health and lower extremity function was found, indicating that a poorer foot health was associated with more difficulties in performing lower extremity‐related actions.

**Conclusion:**

The results suggest that not only are foot problems among people with MS extremely prevalent, but they also impact functional ability. People with MS could benefit from regular rehabilitative care that includes access to podiatric care. Future research is needed to develop and evaluate strategies to support self‐care in lower extremity health among people with MS.

## 1 Introduction

Healthy feet are a precondition for safe movement and independence. Long‐term health conditions, such as neurological, inflammatory or metabolic diseases, may influence foot health and cause significant risk for foot problems. Among neurological diseases, multiple sclerosis (MS) is one of the most common long‐term health problems affecting functional health and functional ability [1]. There are around 2.8 million patients diagnosed with MS worldwide [2, 3] and the incidence of the disease is expected to rise [3, 4]. This estimation indicates the growing need for health and rehabilitation services targeting people with MS. MS is predominantly diagnosed among young, working‐age adult women, but it can affect people of all ages [1]. The main consequences of MS are mobility impairment, balance problems and lower extremity muscle fatigue [5, 6]. Relatively little is known about the level of foot health and possible association with lower extremity function among people with MS [7].

People with MS consider walking to be the most important bodily function [8]. Previous studies have identified many factors associating with lower extremity function among people with MS. Reduced muscle function [6] and lower extremity muscle strength [9] impair walking function. In addition to the common symptoms of MS, other factors such as foot problems may hamper functional activity or increase the risk of falls [10]. The evaluation of the foot health problems of people with MS has been scarce. Based on a systematic review [7], pes cavus and claw toes [11], oedema [12] and altered foot sensation [13] seem to be common among people with MS. These identified foot problems underline the importance of promoting lower extremity function. Moreover, it highlights the need to evaluate perceived foot health and potential association with lower extremity function.

Given the significant impact of MS on functional ability, it is important to investigate foot health and lower extremity function in people with MS. Such knowledge can advance our understanding of the significance of foot health among people with MS and will thus support the development of effective interventions to improve foot health and lower extremity function in people with MS.

The aim of the study was to analyse the level of self‐reported foot health and lower extremity function in people with MS and to identify possible associating factors.

Research questions:

1. What is the self‐reported foot health in people with MS?
2. What is the lower extremity function in people with MS?
3. What factors, if any, associate with the self‐reported foot health among people with MS?

## 2 Materials and Methods

A cross‐sectional survey study design was applied. The study is reported in accordance with the Consensus‐Based Checklist for Reporting of Survey Studies (CROSS) [14].

The survey consisted of the Self‐administered Foot Health Assessment Instrument (S‐FHAI) [15], the Lower Extremity Functional Scale (LEFS) [16] and background variables. The S‐FHAI measures subjective current foot health with 22 items divided into 4 subscales: skin health (12 items), nail health (4 items), foot structure (5 items) and foot pain (1 item). The response scale is dichotomous (no/yes). By summing up the values of individual items, the S‐FHAI produces a total foot health sum variable (range 22–44). Higher values indicate more foot problems, thus poorer foot health. The S‐FHAI has demonstrated satisfactory internal consistency in previous studies (0.60, 0.67, 0.72, respectively [17, 18, 19]). A Rasch analysis has also supported the unidimensionality and item fit of the S‐FHAI [20]. To provide a thorough evaluation of foot pain, items indicating the location (heel, ball of the foot, toes, ankle, shin, knee, hip, thigh) and intensity of the foot pain (slight, moderate, strong or worst imaginable pain) were added.

The Lower Extremity Functional Scale (LEFS) is a patient‐rated lower extremity function measurement instrument [16]. The LEFS consists of 20 questions focusing on physical activities with increasing physical demand, from walking to running on even ground. The 5‐point response scale ranges from 0 (extreme difficulty/unable to perform activity) to 4 (no difficulty). By summing the scores, the scale produces a total score (range 0–80). Lower scores indicate more limitations in lower extremity function [16].

The following background variables were collected: age, time since MS diagnosis, gender, highest education, perceived importance of foot health (5‐point response scale: 1 = very important to 5 = not very important), effect of foot health on daily activities (5‐point response scale: 1 = very much to 5 = very little), perceived knowledge level of foot self‐care (5‐point response scale: 1 = very good to 5 = very poor), and self‐evaluated level of foot health (scale 1 = poor to 10 = best possible foot health).

### 2.1 Data Collection

The data were collected online in April and May 2024 using the Webropol tool in collaboration with a third sector operator, Finnish Neuro Society. Finnish Neuro Society is a national patient association providing guidance, information and short‐term rehabilitation for people with MS or other rare neurological diseases. In Finland there is about in total of 18,000 persons with MS or rare neurological diseases and the majority of the (about 56%) are members of the national patient association [21]. Before data collection a pilot test was done with purposively selected 10 adult patients with MS. The purpose of the pilot test was to test the functionality of the electronic survey tool and ensure the response instructions and items in wording are clear.

For the actual data collection, a total sampling strategy was employed, whereby all eligible adult members of the Society diagnosed with MS were invited to participate. This approach was chosen to maximise coverage and ensure representativeness, as the population size was manageable and clearly defined. Consequently, the sample size was determined by the size of the entire population, rather than by statistical power calculations. A named contact person emailed the invitation, and a link to the study, to all members of the Society who had provided an email address to the system (*N* = 4183). To prevent multiple participation, the survey system employed browser cookies and unique access links. Participants were informed that only one response per individual was permitted. To increase the response rate, two reminders to respond were sent and an announcement about the study was published on the Society's website and social media channels.

### 2.2 Data Analysis

The data were analysed with statistical analysis using SPSS 29.0 software (SPSS Inc., Chicago, IL). Descriptive statistics were used to describe the data (frequency, percentage, range, mean, standard deviation). The dataset contained very few missing values, which were deemed negligible. Therefore, no imputation procedures were applied, and analyses were conducted using available data. Sum variables were formed by counting the item scores and dividing the value by the number of items. This was computed for total foot health level and sum variable levels of skin health, nail health, foot structure and foot pain. Values from the LEFS were summed to get a total score (range 0–80). The scores were interpreted as: 0–20 severe functional limitation, 21–40 moderate functional limitation, 41–60 mild to moderate functional limitation, and 61–80 minimal functional limitation or normal function [22]. To analyse possible differences between age groups the age was classified to three categories (22–44, 45–64, and 65 years and older).

Sample size was large enough (Central limit theorem) to use parametric tests without concerns of normality assumptions. Sample mean has a normal distribution despite of possible skewness in individual variable values. Parametric tests have more power to reveal statistically significant differences or associations between groups and they should be used when it is possible [23].

The associations between self‐reported foot health and lower extremity function were evaluated using the Pearson correlation coefficient. Multifactor Analysis of Variance was used to find the effects of background factors (main effect model: continuous variables used as covariates and categorical variables used as fixed factors). Sidak adjustments for multiple comparisons were used for pairwise comparisons. A statistical significance was detected if the *p*‐value was ≤ 0.05. The reliability of the S‐FHAI was checked with Kuder Richardsson formula.

### 2.3 Ethical Considerations

The study followed good scientific practice [24]. Ethical approval was obtained from the university's ethics committee (14 August 2024, code: 38/2023) and permission to collect the data from the Finnish Neuro Society. The information letter that was emailed to each participant along with the link to the survey covered a description of the purpose of the study, the data collection procedure, anonymity of responses, confidentiality in data handling, analysis and reporting, and the possibility to withdraw the study at any point without any consequences. Once the participants had read the information letter, they indicated their informed consent electronically and then proceeded to respond to the survey.

## 3 Results

### 3.1 Description of Participants

A total of 969 people with MS responded to the survey (response rate 23%, Table 1). Their mean age was 53 years (range 24–82, SD 12) and they were predominantly women (*n* = 771, 80%). The time since MS diagnosis varied from 6 months to 72 years with an average of 16 years (SD 10). As their highest level of education, the majority of participants had a degree from a university of applied sciences (*n* = 380, 39%) or from a vocational school (*n* = 253, 26%). The participants perceived the importance of foot health as very important (*n* = 618, 64%). They also perceived that their current foot health status affected their daily activities much (*n* = 328, 34%) or very much (*n* = 362, 38%). The majority of the participants considered their knowledge levels of foot self‐care to be very good (*n* = 362, 38%) or good (*n* = 328, 34%). One in 10 participants (*n* = 95, 10%) had been on sick leave due to foot problems. Overall, the participants evaluated their current level of foot health on a scale 1–10 as 7 (range 1–10, SD 2.2) indicating moderate foot health.

**TABLE 1:** Participants' (*n* = 969) background information.

Background variable	*n*	(%)	Mean, range, SD	Median
Age	968		53, 24–82, SD 12	53
Duration of MS	968		16, 0.5–72, SD 10	15
Gender				
Women	771	80		
Men	186	20		
Highest education				
Elementary school	47	5		
Vocational school	253	26		
High school	46	5		
University of applied sciences	380	39		
University	238	25		
Perceived importance of foot health				
Very important	618	64		
Important	302	31		
Somewhat important	37	4		
Not very important	5	1		
Effect of foot health to daily activities				
Very much	362	38		
Much	328	34		
Neither too much nor too little	142	15		
Little	64	7		
Very little	70	7		
Perceived knowledge level of foot self‐care				
Very good	362	38		
Good	328	34		
Neither good nor poor	142	15		
Poor	64	7		
Very poor	70	7		
Sick leave from work due to foot problems				
Yes	95	10		
Self‐evaluated level of foot health	965		6.6, 1–10, SD 2.2	7

### 3.2 Self‐Reported Foot Health in People With MS

The participants had experienced multiple foot health problems (Table 2). Overall, the mean value for total foot health was 29 (range 22–40), indicating a relatively poor level of foot health with moderate amount of foot problems.

**TABLE 2:** Self‐reported foot health in people with MS (*n* = 969).

Variable	*n*	%
Foot skin		
Skin breaks or maceration between toes	140	15
Dry skin	705	73
Fissures in the heel	373	39
Corns or calluses	488	50
Verrucae	97	10
Blisters	33	3
Oedema	423	43
Sweating feet	235	24
Burning feet	258	27
Cold feet	626	65
Leg cramps	589	61
Foot ulcers	12	1
Toenails		
Ingrown nail	143	15
Thickened nail	495	51
Colour changes in the nails	328	34
Fungal infection of the nails	79	8
Foot structure		
Hallux valgus	239	25
Taylor's bunion	187	19
Hammer toe	234	24
Low foot arch	309	32
High foot arch	96	10
Foot pain	568	59

*Note:* Self‐administered Foot Health Assessment Instrument (S‐FHAI, Stolt et al. [15]) Stolt.

Regarding foot skin health, the most common problems were dry skin (73%), cold feet (65%), leg cramps (61%) and corns or calluses (50%). Concerning toenail health, thickened toenails (51%) and colour changes in the nails (34%) were most prevalent. In the area of foot structure, hallux valgus (25%) and hammer toes (24%) were most common.

**FIGURE 1:**
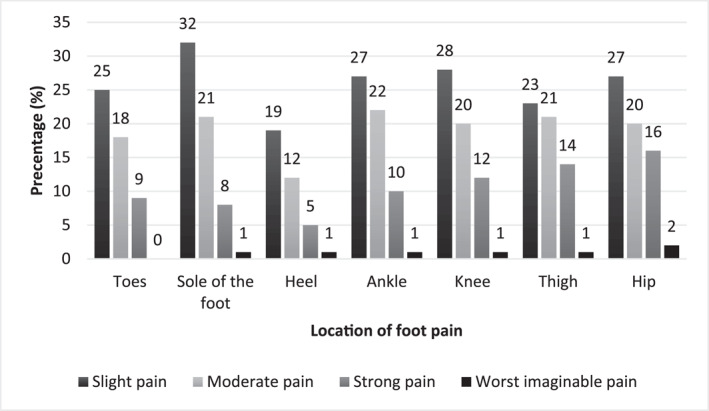
Location and prevalence of foot pain in people with MS (*n* = 969).

Foot pain was experienced by slightly over half of the participants (*n* = 550, 59%, Figure 1). The pain was most prevalent in the hip (intensity: slight 27%, moderate 20% and strong pain 16%), sole of the foot (slight 32%, moderate 21% and strong pain 8%), knee (slight 28%, moderate 20%, strong 12%) and ankle (slight 27%, moderate 22%, strong pain 10%).

### 3.3 Lower Extremity Function in People With MS

On average, mild to moderate lower extremity‐related functional limitation was reported by the participants (mean 51, SD 22, range 0–80) (Table 3). Severe or moderate functional limitation was experienced by 12% (*n* = 121) and 21% (*n* = 200), respectively. Mild to moderate functional limitation was reported by 26% (*n* = 254) of participants. Many of the participants had minimal functional limitation or normal function (*n* = 394, 41%).

**TABLE 3:** Lower extremity function reported by patients with MS (*n* = 969).

Activity	*N*	Extreme difficult or unable to perform activity	Quite a bit of difficulty	Moderate difficulty	A little bit of difficulty	No difficulty
		*n* (%)	*n* (%)	*n* (%)	*n* (%)	*n* (%)
Work, housework or school activities	967	38 (4)	91 (9)	169 (18)	306 (32)	363 (38)
Usual hobbies, recreational or sporting activities	967	53 (6)	133 (14)	207 (21)	325 (34)	249 (26)
Getting into or out of the bath	941	150 (16)	62 (7)	94 (10)	167 (18)	468 (50)
Walking between rooms	968	67 (7)	38 (4)	87 (9)	208 (22)	568 (59)
Putting on shoes or socks	967	31 (3)	65 (7)	129 (13)	303 (31)	439 (45)
Squatting	967	128 (13)	78 (8)	137 (14)	239 (25)	385 (40)
Lifting an object	965	49 (5)	68 (7)	93 (10)	226 (23)	529 (55)
Light activities	964	23 (2)	46 (5)	112 (12)	253 (26)	530 (55)
Heavy activities	967	159 (16)	161 (17)	158 (16)	234 (24)	255 (26)
Getting into or out of a car	967	42 (4)	64 (7)	135 (14)	287 (30)	439 (45)
Walking 2 blocks	963	157 (16)	89 (9)	76 (8)	155 (16)	486 (51)
Walking 1 km	962	231 (24)	71 (7)	92 (10)	153 (16)	415 (43)
Going up or downs 10 stairs	968	130 (13)	110 (11)	116 (12)	224 (23)	388 (40)
Standing for 1 h	966	260 (27)	105 (11)	153 (16)	213 (22)	235 (24)
Sitting for 1 h	965	19 (2)	42 (4)	86 (9)	273 (28)	545 (57)
Running on even ground	956	444 (46)	93 (10)	104 (11)	116 (12)	199 (21)
Running on uneven ground	961	488 (51)	104 (11)	97 (10)	121 (13)	151 (16)
Making sharp turns while running fast	961	475 (49)	103 (11)	98 (10)	125 (13)	160 (17)
Hopping	963	365 (38)	137 (14)	120 (13)	145 (15)	196 (20)
Rolling over in bed	967	30 (3)	65 (7)	96 (10)	218 (23)	558 (58)

*Note:* The Lower Extremity Function Scale (LEFS, Binkley et al. [16]) Binkley.

For the majority of participants, running on even (*n* = 444, 46%) or uneven (*n* = 488, 51%) ground and making sharp turns while running fast (*n* = 475, 49%) was extremely difficult or not possible at all. Similarly, hopping (*n* = 365, 38%), standing for 1 h (*n* = 260, 27%) and walking 1 km (*n* = 231, 24%) caused extreme difficulties for the participants.

### 3.4 Associating Factors to the Self‐Reported Foot Health Among People With MS

The self‐reported foot health among people with MS associated with gender, being on sick leave due to foot problems, perceived knowledge levels of foot self‐care, self‐evaluated level of foot health and lower extremity function.

A statistically significant difference in foot health scores was found between genders (*F*(1, df) = 1966.28, *p* = 0.019, partial *η* ^2^ = 0.006) with women exhibiting poorer foot health than men. Poorer foot health was also significantly associated with being on sick leave from work (*F*(1, df) = 15.610, *p* < 0.001, partial *η* ^2^ = 0.170). Participants who perceived their foot self‐care knowledge as very good or good had better foot health (*F*(1, df) = 5.885, *p* < 0.001, partial *η* ^2^ = 0.026) compared to those who reported poor knowledge. Additionally, participants who self‐evaluated their foot health as higher had significantly better foot health scores (*F*(1, df) = 25.602, *p* < 0.001, partial *η* ^2^ = 0.028). There was a significant negative correlation (*r* = −0.259, *p* < 0.001) indicating that better foot health was associated with better lower extremity function.

The effect of age group on foot health was not statistically significant (*F*(2, 965) = 1.45, *p* = 0.236). The mean difference in lower extremity function between young and middle‐aged participants was 15.74 (*p* < 0.001, 95% CI [12.29, 19.19]), and young and older aged was 24.32 (*p* < 0.001, 95% CI [19.63, 29.00]). Similarly, the mean difference in lower extremity function between participants with age between 45 and 64 and older (age 65 and higher) individuals was 8.59 (*p* < 0.001, 95% CI [4.15, 13.02]).

## 4 Discussion

People with MS had many foot health problems and they reported mild to moderate lower extremity‐related functional limitation. Associating factors to the self‐reported foot health among people with MS were gender, being on sick leave due to foot problems, perceived knowledge level of foot self‐care, self‐evaluated level of foot health and lower extremity function.

Foot health was found to have a weak correlation with lower extremity function in people with MS. The poorer the level of foot health a person had, the more difficulties they had experienced in performing lower extremity‐related actions. Even the correlation was weak, it demonstrates the importance of foot health as one part in lower extremity health. Lower extremity function is frequently impaired in people with MS due to motor weakness, spasticity and balance difficulties, which can significantly affect mobility and quality of life [25]. Walking impairment is one of the most commonly reported symptoms [26]. This finding underlines the importance to develop multiprofessional rehabilitative services for people with MS.

High prevalence of foot pain, structural deformities and mobility limitations among people with MS suggest that fear‐avoidance may be a relevant psychosocial factor [27]. People with MS often experience elevated levels of kinesiophobia or fear of movement due to pain of muscle stiffness which can exacerbate functional limitations and reduce participation in physical activity [25]. Similarly, people with MS experience reduced quality of life and even depression [28] which may be a hindering factor to physical activity. Therefore, assessing the psychosocial dimensions is important to gain comprehensive understanding of the mobility‐related challenges faces by the people with MS. Furthermore, future studies could benefit of assessing the relationship between foot health, physical activity and depression.

The number of self‐reported foot problems was high among people with MS. The most common foot problems were foot pain, skin and nail problems, and foot structural problems. These problems are similar to those of the general population [29, 30, 31]; however, their prevalence seems to be higher in people with MS. Previous research has identified many other foot health conditions such as pes cavus, claw toes oedema, and altered foot sensation, which may contribute to pain, instability and reduced self‐care ability [7]. However, research on foot health in MS remains limited, and factors associated with self‐reported foot health—such as gender, perceived knowledge of foot care and functional ability—are not yet fully understood.

Participants with higher levels of perceived knowledge of foot self‐care had better foot health. This suggests that people with MS are keen to know how to self‐care for their feet. To maintain functional health, people with MS would gain advanced if they have access to preventive care, patient education and support for foot self‐care. The majority of the identified problems could be treated by a podiatrist [32], but access to public podiatric care may be targeted to other patient groups, such as those with diabetes mellitus. In order to promote functional health among people with MS, it would be important to create and develop supportive structures for people with MS to help them to promote their foot health. This could be done, for example, via third sector associations who could deliver digital short‐term educational sessions and create easy‐to‐access websites about foot health for people with MS.

Some participants reported having been on sick leave due to foot problems. Being on sick leave is associated with a number of foot problems. This finding is significant as many patients with MS are young, working‐age adults [1]. Preventative activities in collaboration with, for example, occupational health care are crucial for promoting and supporting the working ability and functional health of people with MS.

The outcomes of podiatric care were not evaluated in this study. However, the results reveal significant amount of foot health problems. These problems may indicate that people with MS could benefit from regular podiatry services. Therefore, future research could focus on analysing the outcome of podiatric services to foot health in people with MS. As an implication, people with MS could benefit from regular rehabilitative activities provided by multiprofessional teams to develop the lower extremity health. MS as a long‐term health condition is known to have relapsing and remitting stages where physical functional ability may vary. However, it is important to assess and care for lower extremity health as lower extremities are often the first site of the body where deficits in muscle activity and power emerge due to sedentary behaviour [33]. To support muscular capacity in people with MS, multiprofessional teamwork with physiotherapists, podiatrists and exercise experts are needed.

### 4.1 Strengths and Limitations

This study has some strengths and limitations. The study was a cross‐sectional survey study with no control group nor controlled for confounding (e.g., BMI), therefore inferences are limited to correlation restricting any causation. Future research could therefore investigate the association of physical limitations [34] or body weight with foot health and functional activity.

Data collection with electronic survey and participant recruitment via patient association might have caused some selection bias. Many of people with MS in Finland are members of the national patient association, however still the electronic survey might have reached only those who are digitally literate.

Response rate was only 23% which may reflect nonresponse bias. Individuals who are more engaged to their care or more concerned about their foot health may be overrepresented in the sample and therefore limit the generalisability of the results. The size of the sample allowed for conducting robust statistical analysis and detecting associations in foot health and background factors.

The S‐FHAI is a self‐administered subjective instrument. Subjective evaluation may have led to an over‐ or underestimation [35] of the actual foot health level or may have increased the likelihood of recall or social desirability response bias [36]. However, despite possible errors, it is of utmost importance to collect individual's subjective observations of their own feet. The S‐FHAI has been used in previous studies [17, 18, 19], and it demonstrated acceptable internal consistency in this study (Kuder–Richardsson formula 0.61). Internal consistency of the instrument is below the recommended criteria (0.70) [37] warranting further research to confirm the results. Future studies could benefit of using additionally other validated foot‐specific patient outcome measures, such as the Manchester–Oxford Foot Questionnaire (MOxFQ) [38] or Foot Health Status Questionnaire (FHSQ) [39] to assess the completeness of lower extremity health.

## 5 Conclusion

This study demonstrates that people with MS have many self‐reported foot problems and they experience mild to moderate lower extremity‐related functional limitations. Foot health among people with MS associated with gender, being on sick leave due to foot problems, perceived knowledge levels of foot self‐care and self‐evaluated level of foot health. Moreover, weak but significant correlation between foot health and lower extremity function was found, indicating that a poorer foot health was associated with more difficulties in performing lower extremity‐related actions.

The results underline that people with MS could benefit of the provision of targeted foot health education and podiatric care. This kind of support could help them to manage their foot health and motivate them to care for their feet. These findings highlight the importance of further investigating foot health as a potentially modifiable factor influencing functional mobility in people with MS. Future research should explore causal pathways between foot health and lower extremity function, examine interventions aimed at improving foot care knowledge and self‐management, and consider longitudinal designs to assess changes over time. Additionally, qualitative studies could provide deeper insights into how foot problems impact daily functioning and quality of life in this population.

## Author Contributions

**Minna Stolt:** conceptualization, data curation, resources, project administration, funding acquisition, investigation, writing – original draft, writing – review and editing. **Maria Eränen:** conceptualization, data curation, investigation, formal analysis, writing – original draft, writing – review and editing. **Jouko Katajisto:** data curation, formal analysis, methodology, validation, writing – original draft, writing – review and editing. **Riitta Rosio:** conceptualization, methodology, supervision, writing – original draft, writing – review and editing.

## Ethics Statement

Ethical approval was obtained from the university's ethics committee (14.8.2024, code: 38/2023).

## Conflicts of Interest

The authors declare no conflicts of interest.

## Supporting information


Supporting Information S1

## Data Availability

The data that support the findings of this study are available from the corresponding author upon request. The data are not publicly available due to ethical restrictions.
